# Epigenetic impacts of maternal tobacco and e-vapour exposure on the offspring lung

**DOI:** 10.1186/s13148-019-0631-3

**Published:** 2019-02-19

**Authors:** Razia Zakarya, Ian Adcock, Brian G. Oliver

**Affiliations:** 1 Respiratory Cellular and Molecular Biology, Woolcock Institute of Medical Research, The University of Sydney, Sydney, Australia; 2 School of Life Sciences, University of Technology Sydney, Sydney, Australia; 3 Airway Diseases Section, National Heart and Lung Institute, Imperial College London, London, UK; 4 Biomedical Research Unit, Section of Respiratory Diseases, Royal Brompton and Harefield NHS Trust, London, UK

**Keywords:** Epigenetics, Airway, Lung development, Asthma, COPD, E-cigarette, Tobacco, Foetal

## Abstract

In utero exposure to tobacco products, whether maternal or environmental, have harmful effects on first neonatal and later adult respiratory outcomes. These effects have been shown to persist across subsequent generations, regardless of the offsprings’ smoking habits. Established epigenetic modifications induced by in utero exposure are postulated as the mechanism underlying the inherited poor respiratory outcomes. As e-cigarette use is on the rise, their potential to induce similar functional respiratory deficits underpinned by an alteration in the foetal epigenome needs to be explored. This review will focus on the functional and epigenetic impact of in utero exposure to maternal cigarette smoke, maternal environmental tobacco smoke, environmental tobacco smoke and e-cigarette vapour on foetal respiratory outcomes.

## Background

Foetal lung organogenesis is an extensive and multi-stage process, commencing with the development of the lung bud by the 4th gestational week, with lobar and vascularised subsegmental branching occurring by the 6th week [1]. Genesis of conducting airways, with airway cartilage, smooth muscle, mucous glands and epithelial cell differentiation commences as early as gestational week 7 [1]. Completion of a full-term pregnancy allows for formation of true alveoli and maturation of surfactant in Type II epithelial cells [1, 2], allowing for healthy gas exchange. Upon delivery, lung development will continue postnatally, with significant alveolar growth occurring during the first 2 years of life [3] and into adolescence [4]. This protracted period of development, commencing in utero and continuing into adolescence, makes the pulmonary system particularly vulnerable to environmental insults affecting normal lung development. Harmful exposures during development can alter the course of healthy lung development and set the child on a trajectory making them more vulnerable to disease [5–7].

Asthma and chronic obstructive pulmonary disease (COPD) are diseases of the airway, wherein patients experience common symptoms such as shortness of breath, cough and wheeze, and share some similar pathological changes collectively termed airway remodelling. What sets them apart at a functional level is the age of onset of symptoms, etiological causes, progression of the disease and response to existing therapeutics.

Asthma is a heterogenous disease experienced by 235 million people worldwide [8] and is the most prevalent chronic disease in developed countries. Asthma typically develops early in life with patients experiencing symptoms during an exacerbation episode known as an ‘asthma attack’, which typically responds well to bronchodilators and can be controlled using corticosteroids. Overall, asthmatic mortality rates have fallen but deaths during asthma attacks subsist, with higher prevalence in the elderly [9]. Atopy is common in asthma, mediated by CD4+ Th2 cells and infiltration of mast cells and eosinophils in the airway walls. Inflammation and increased smooth muscle bulk comprise airway wall remodelling in asthma, causing airway obstruction [9, 10].

COPD is the fourth most common cause of death worldwide with prevalence increasing in concert with the ageing population [11, 12]. In contrast to the age of onset in asthma, COPD—except anti-α-trypsin COPD—develops later in life. COPD patients generally show a limited response to corticosteroids and upon manifestation of the disease, lung function progressively declines until death or transplantation. Inflammation in small airway walls of COPD patients is mediated by CD8+ Tc1 cells, consists of neutrophils and macrophages [9, 10] and is most prevalent in peripheral airways [13]. In conjunction with small airway obstruction, COPD patients may experience emphysema, which manifests as loss of alveolar space. Pathologically, patients can be clustered into predominantly experiencing either small airway obstruction or emphysematous destruction [9].

Both asthma and COPD have an inherited component, but the aetiology and risk factors for the two are different. Typically, asthma is an allergic disease and COPD is the result of inhalation of noxious gases; however, there is considerable overlap of the two diseases, and in some cases, asthma and COPD can co-exist and asthma can progress into COPD. The differences between COPD and asthma are attributed to different gene environment interactions and different genetic risk factors. Pathologically the two diseases are distinct, for example differing inflammatory profiles and sites of inflammation within the airway wall [9, 14], but asthmatics and COPD patients both experience obstruction of the airways. A useful diagnostic tool for airway obstruction is spirometry, wherein patients’ forced expiratory volume in 1 second (FEV_1_) demonstrates how quickly a patient can expel air from their lungs. A lower FEV_1_ indicates greater airway obstruction. The main spirometric difference is that asthma has reversible airway obstruction, but COPD has incomplete reversal of airway obstruction. However, spirometry alone cannot differentially diagnose the two diseases. In severe forms of asthma, for example asthma with fixed airflow limitation, lung physiology can resemble COPD, and similarly patients with COPD can be highly responsive to bronchodilators.

This review will focus on the epigenetic impact of specific environmental insults such as environmental tobacco smoke (ETS), maternal exposure to ETS (METS), maternal use of tobacco smoke (MTS) and maternal e-cigarette vapour (MEV) exposure on the offspring’s lung development and function, with a focus on asthma and COPD.

### Epigenetics in asthma and COPD

Studies have shown that family history of COPD is a risk factor for manifestation of the disease [15, 16]. Similarly, siblings and first-degree relatives of asthmatics are often affected with lower FEV_1_ [17, 18], thereby suggesting a heritability factor in asthma and COPD. The absence of a correlation between findings of a COPD or asthma SNP in genome-wide associations studies (GWAS) suggests that the hereditary effect is likely established at the epigenomic level rather than genomic and might have greater impact on gene expression in cells at the site of disease [19].

Epigenome-wide association studies (EWAS) have found that leukocytes from COPD patients have 349 differentially methylated CpG sites compared to those from non-COPD smokers [20]. A similar study using small airway epithelial cells found 1260 differentially methylated CpGs related to COPD [21]. DNA methylation status at the promoter of *GATA4* measured in sputum samples has been associated with impaired lung function [22, 23] and health outcomes in COPD [22]. Whilst augmented mRNA expression of *DEFB1*, a gene associated with COPD [24], has been attributed to trimethylation of H3K4 [25].

The balance of Type 1 helper T-cells (Th1) and Type 2 helper T-cells (Th2) is crucial in the development of atopic asthma [26]. Epigenetic changes, such as methylation at the interferon-γ (*Ifn-γ*) promoter, have been associated with skewing naïve T-cells towards an atopic Th2 phenotype [27]. Murine models of asthma have shown that genetic components involved in transcription of Th2 cytokine, IL-13, are regulated by DNA methylation and miRNAs with predicted targets essential in allergic airways disease [28].

The innate immune system is naturally plastic and therefore particularly vulnerable to epigenetic modifications. Further, aberrant accumulation of leukocytes such as neutrophils and eosinophils has been implicated in both asthma and COPD [9] suggesting that dysregulated epigenetic modulation of these cells could contribute to disease pathology. A study using bronchoalveolar lavage (BAL) macrophages from patients with COPD found lower expression of *HDAC2* mRNA and showed decreased histone deacetylase (HDAC) activity in smokers that correlated with significantly higher levels of IL-1β and TNFα [29]. There was an altered ability of the BET mimic JQ1 to suppress specific cytokine gene expression in COPD BAL macrophages [30] which together demonstrate that epigenetic changes contribute to disease pathology. For a comprehensive review on epigenetics in airways disease, it is recommended to read Durham et al [31].

### Functional and epigenetic outcomes of maternal tobacco smoke (MTS), maternal environmental tobacco smoke (METS) and environmental tobacco smoke (ETS) exposure

Although awareness campaigns have led to a general decline in smoking rates across the world, MTS is an ongoing issue [32, 33]. Rates vary widely between countries, with some EU nations as low as 5% (Sweden, Austria, Switzerland) and others as high as 40% (Greece) [34–36]; in the US 10.7% of mothers smoke during the last trimester [33]. Together, these data demonstrate that maternal smoking is a worldwide problem. Maternal tobacco use is not the only means of foetal tobacco exposure with epidemiological studies reporting up to 50% of women in China are exposed to ETS while pregnant [37]. Further, it is estimated that the aforementioned MTS and ETS exposure rates do not accurately reflect the true extent of the problem as smoking parents have been shown to falsely report their habit [38] and 50% of smokers continue to smoke throughout their pregnancy [39].

Studies have quantified levels of cotinine in amniotic fluid of pregnant smokers and blood from neonates exposed to MTS [40, 41], confirming that nicotine can cross the placenta in utero [40, 42]. An investigation of nicotine exposure in neonates found cotinine levels comparable to that observed in active smoking adults [43, 44]. It is presumed that the antenatally exposed infant will continue to be exposed to nicotine postnatally through ETS exposure and breast milk [45, 46] with 40% of children reportedly exposed to ETS [47]. Studies have found a positive correlation between concentration of nicotine in maternal blood and foetal growth retardation [48].

Harmful effects of MTS on lung development have been detected early on with a slower pace of septal growth, subsequent alveolarisation [49, 50], and foetal lung size of MTS-exposed babies reduced by the 33rd gestational week [51]. Mothers continuing to smoke during pregnancy have a 25% higher likelihood of preterm labour [52], causing a disruption of healthy lung organogenesis leading to aberrant development [53].

MTS exposure also increases risk of asthma [54, 55] and wheeze [54, 56] in the offspring, with paternal smoking being an additive risk [55]. Negative respiratory outcomes for infants exposed to MTS include irregular tidal breathing patterns, decreased passive respiratory compliance, and decreased forced expiratory flows [51, 57], with decreased lung function persisting into adolescence [55, 57] and early adulthood [58, 59]. Paternal smoking during puberty, when spermatogonia are developing, increases the risk for asthma in offspring [60], thereby demonstrating that parental smoking behaviour has a long-term effect on respiratory outcomes in the offspring.

Exposure to ETS significantly decreases FEV_1_ [61, 62] and is an independent risk factor for developing asthma [63]. Asthmatic children exposed to ETS have more severe asthma [64] and frequent exacerbations requiring hospitalisation [65] and tend to have slower recoveries than those not exposed to ETS [66]. Indeed, urinary cotinine levels positively correlate with ETS exposure levels and the severity of asthma exacerbations [67] and higher blood cotinine concentrations are linked to bronchial hyperresponsiveness [68]. Removing ETS from an asthmatic child’s environment has shown positive health outcomes by lessening symptoms [69]. Women exposed to ETS during childhood were twice as likely to develop COPD whilst men showed a slightly increased risk of reduced lung function when compared with those not exposed to ETS during childhood [70]. Childhood ETS exposure combined with previous MTS exposure has been shown to have compounding effects that leave the offspring more vulnerable to harmful effects of active smoking and decline in lung function [58, 71]. The effect of MTS and ETS on COPD patients’ outcomes persists long into their lives, with adult patients of smoking mothers having significantly lower FEV_1_ than those of non-smoking mothers [72].

Investigations into epigenetic aberrations in human airway cells exposed to tobacco smoke found small airway epithelial cells experience dose-dependent changes in histone acetylation and methylation, alongside decreased expression of DNA methyltransferases (DNMT) [73]. Tobacco smoke-exposed H292 cells, derived from human lung epithelia, showed augmented expression of genes for enzymes involved with chromatin modifications, such as the histone deacetylase (HDAC), *HDAC2*, and the histone acetyltransferase (HAT), *Myst4*, within 60 min of exposure to tobacco smoke extract with expression of other HATs and HDACs upregulated at the 24-h time point [74]. Exposure of human bronchial epithelial cells to the vapour phase of tobacco smoke, rather than a tobacco smoke extract, found that tobacco smoke induces acetylation at H3K27 and demonstrate that these changes have a downstream effect on transcription of genes related to stress responses [75].

COPD is a known risk factor for lung cancer and the latter is also associated with an altered epigenome, and several specific changes in miRNA expression, histone modifications and DNA methylation profiles have been reported in lung cancer and even proposed as biomarkers of disease [76]. For example, the methylation status of PGAM5 in human sperm cells is altered by cigarette smoking which affects its expression [77]. PGAM5 expression was dysregulated in epithelial cells and specific macrophage subtypes of COPD patients with lung cancer with the latter associated with mortality [78].

Epidemiological evidence supports the notion that the effects of MTS are heritable with further generations continuing to manifest poor respiratory outcomes. Grandmaternal smoking has been shown to affect the grandchild’s lung development [60, 79] and increase the risk of asthma independent of maternal smoking [80–82]. Furthermore, MTS exposure experienced by the father in utero has been shown to affect the respiratory outcome of his daughter, independent of his smoking habits [83]. Murine models confirm the direct effects of MTS on the offspring with in utero smoke exposure decreasing lung volume [84, 85] and increasing airway resistance [85] and provide insights into the mechanisms underlying these changes. The developmental differences are evident in MTS-exposed mice offspring with significantly lower lung weights [86] and increased ASM layer thickness and collagen deposition upon allergen challenge with HDM compared to those exposed to ambient air [87]. An intergenerational murine model demonstrates that METS exposure lead to increases in airway hyperactivity, airway resistance and decreases in lung compliance in offspring, which was then passed down to the next generation in the absence of METS exposure [88]. Similarly, allergen challenge elicited an ameliorated atopic response demonstrated by eosinophilia and significantly higher IL-13 levels in two subsequent generations when compared to the progeny of ambient air exposed animals [88]; METS exposure and allergen challenge were shown to deregulate miR-130, miR-16 and miR-221 exposure and are postulated as the epigenetic mechanism modulating the augmented IL-13 response induced by METS exposure [88].

Cigarette smoke constituents have been detected in both the placenta and cord blood [43, 44] of newborns and MTS exposure has been shown to cause changes in global DNA methylation [89–93] and alter miRNA levels in germline cells [94]. Hence, there is no question that MTS exposure alters the foetal epigenome. The effects of aberrant DNA methylation patterns in cord blood and placenta are demonstrated by tissue-specific DNA methylome analyses showing that MTS can induce specific changes to DNA methylation within the placenta in genes crucial to foetal growth and development [92, 95]. Further, blood DNA methylation changes have been associated with lower FEV_1_ [96] and have been shown to persist into childhood and adolescence [90, 97–101], demonstrating that epigenetic modulations induced by MTS have long-lasting effects on offspring’s lung function (Table 1). Various studies have shown that DNA methylation changes caused by MTS occur at loci specific to established outcomes of maternal smoking such as reduced foetal growth and wheeze [102, 103].

**Table 1:** Summary of respiratory function-specific epigenetic changes in the offspring categorised by exposure

Epigenetic changes induced by MTS	
Altered global DNA methylation	Whole blood, cord blood, placenta [89–93]
Genes associated with foetal growth—*LINE-1, AluYb8, IGF2DMR, Igf1R, Igf2*—differentially methylated	Placenta, cord WBC, cord blood, murine lung [95, 102, 103, 109, 110]
COPD candidate gene in GWAS, *DPP10*, hypomethylated	Foetal lung [104–106]
Genes associated with detoxification of tobacco smoke, *CYP1A1* and *AHRR*, show altered methylation	Placenta, cord blood [91, 107]
miRNA involved in transcription of *Igf1* upregulated	Murine lung [86]
Epigenetic changes induced by METS	
*IL-4* and *IL-13* hypomethylated at promoter region	Murine lung [118]
miR-155-5p, miR-21-3p and miR-18a-5p positively correlate with Th2 cytokines	Murine lung [118]
Epigenetic changes induced by MEV	
Global DNA hypermethylation	Murine lung [130]

EWAS findings have shown MTS induced altered methylation of *DPP10* [104], a candidate gene identified in GWASs [105, 106], in human foetal lung tissue. Genes playing a role in attenuating the harmful effects of tobacco smoke and its toxic constituents, such as *CYP1A1* [91] and *AHRR* [107], are modulated by DNA methylation and have been shown to be altered by MTS exposure. Immune cells from active smoking adults and cord blood from neonates exposed to MTS both show differential methylation of *CYP1A1* and *AHRR* promoter regions compared to non-smoke-exposed subjects [91, 108]. MTS exposure has been shown to cause demethylation of the promoter region for receptor of insulin-like growth factor 1 (*Igf1R*) in the murine lung [109] and methylation of insulin-like growth factor 2 (*Igf2)* in human cord blood [110], which both play an important role in lung development and can contribute to asthma later in life. Interestingly, the differentially methylated regions in *Igf1R* and *Igf2* induced by MTS have been shown to be sex dependent, with the former only evident in females and the latter males [109, 110]. Taken together with studies showing MTS exposure affecting organs differently [111], the findings fortify the requirement for specificity in epigenetic investigations as stimuli causing demethylation in one organ or gender can have inverse effects in another.

**Fig. 1:**
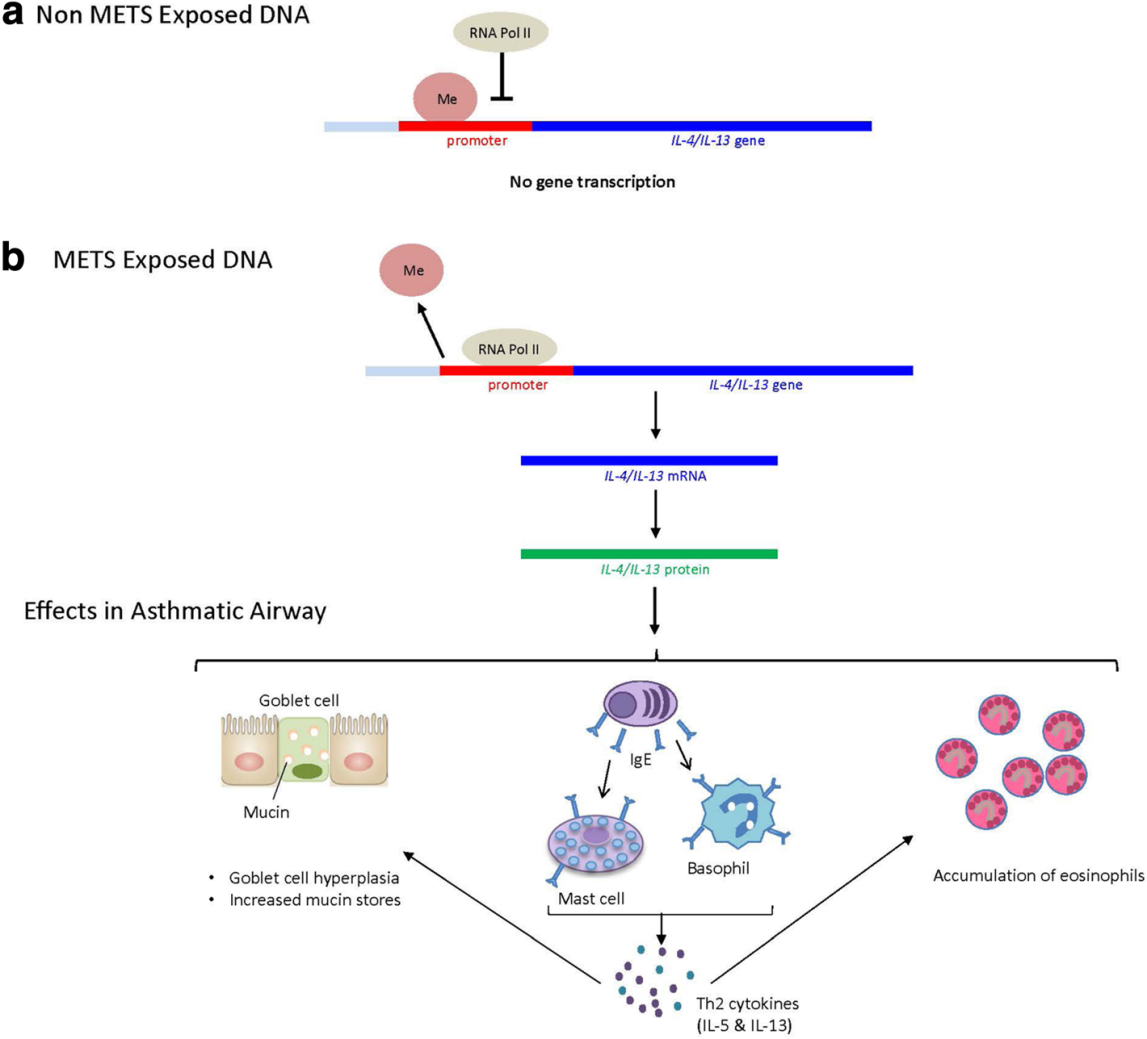
Effect of *IL-4* and *IL-13* promoter region hypomethylation. **a** Methylation (Me) inhibits binding of RNA Polymerase II (RNA Pol II) to gene promoter region, thereby suppressing gene transcription. **b** METS exposure demethylates *IL-4* and *IL-13* promoter region in offspring [114, 116], allowing for RNA Pol II to commence mRNA transcription, leading to IL-4 and IL-13 protein translation; therein contributing to pathological changes in the airway wall leading to goblet cell hyperplasia, increased mucin stores, promotion of IgE production, and accumulation of eosinophils, mast cells and basophils. Mast cells and basophils further produce Th2 cytokines IL-5 and IL-13, further perpetuating the airway inflammation

Further investigations have shown that MTS exposure dysregulated 133 miRNAs expressed in foetal murine lungs, some of which played a role in transcription of *Igf1* which was significantly increased in female offspring [86]. The authors validated these findings in humans by showing increased *Igf1* mRNA expressed from leukocytes of school-aged children exposed to MTS [86], demonstrating that the mechanism is conserved between species and persists beyond infancy. METS alters lung structure [112] and lowers birth weight in murine models of exposure [88, 113]. Upon allergen challenge, METS-exposed murine offspring express significantly higher levels of Th2 cytokines in BAL fluid and lung, lung eosinophilia and airway hyperreactivity when compared to offspring exposed to ambient air antenatally [114, 115] which corresponds with strong hypomethylation at the *IL4* and *IL13* promoters [114]. Augmented expression of IL-13 in airways of METS-exposed murine offspring correspond with demethylation at the *IL13* promoter [116] demonstrating alterations to DNA methylation induced by METS exposure contribute to pathology in allergic asthma. Specific miRNAs are implicated as regulators of the Th1/Th2 balance with ablation of miR-21 expression significantly augmenting expression of Th1 cytokine IFNγ and ameliorating expression of Th2 cytokine, IL-4 [117] in mice (Fig. 1). A study of allergen-challenged mice exposed to METS found a strong correlation between miR-155-5p, miR-21-3p and miR-18a-5p and expression of Th2 cytokines in BAL [118], implicating miRNAs in the modulation of METS-induced atopy in offspring. These findings are compelling when conjoined with the previously discussed study by Singh et al. [88] implicating miRNAs in METS-induced augmented IL-13 production.

### Functional and Epigenetic effects of MEV exposure

The negative health impacts of cigarette smoking are well documented and agreed upon. As cigarette consumption declines, an opening in the market has formed. In response, established tobacco companies and entrepreneurs alike have flooded the market with new nicotine delivery devices. The most successful thus far being the e-cigarette. Briefly, an e-cigarette is a hand-held device comprised of a reservoir for an “e-liquid” and a heating element connected to a battery. Upon use, the e-liquid passes through the heating element, forming an “e-vapour” to be inhaled by the user. Unlike a cigarette, there is no combustion in an e-cigarette and it is subsequently marketed as a “healthier” alternative to cigarette smoking. However, the declaration of healthiness is premature as the effects of long-term e-cigarette use and indirect exposure to e-vapour remain to be elucidated. The illusion of a healthier alternative leaves the population at risk of enduring damaging effects with at-risk groups being the most vulnerable. It has been reported that pregnant women have started to use e-cigarettes during pregnancy at increasing rates [119].

The basic composition of an e-liquid is a mixture of propylene glycol, glycerol and flavourings, which may include nicotine but some e-liquids contain no nicotine [120]. Notwithstanding coming under the jurisdiction of the EU Tobacco Products Directive in May 2016, e-liquid compositions continue to vary widely, and studies have identified discrepancies in actual versus reported nicotine concentrations [121, 122]. Independent analyses have detected harmful compounds such as phthalates, diacetyl and acrolein in e-liquids [122–124]. Whilst indoor air quality studies have found that levels of aerosolised polycyclic aromatic hydrocarbons (PAHs), formaldehyde, acetaldehyde, acrolein and particulate matter ≤ 2.5μm [120, 122] are significantly increased when e-cigarettes are used indoors.

As established, the ingredients in an e-liquid vary widely, with some shown to be capable of epigenetic modifications. An in vitro experiment using EA.hy926 cells found that DNMT3b transcript was decreased following acrolein exposure [125]. Maternal exposure to benzylbutylphthalate (BBP) caused global DNA hypermethylation in CD4+ T cells of the exposed dam and to a greater extent in her offspring in a murine model of exposure [126]. This hypermethylation significantly correlated with attenuated expression of the GATA-3 repressor zinc finger protein 1 (*Zfpm1)—*a gene that represses GATA-3 mediated Th2 cell development—thereby promoting the Th2 phenotype. The authors further validated the link between maternal urinary BBP metabolite levels and *Zfpm1* in humans using whole blood samples from 4-year-old children in the lifestyle and environmental factors and their influence on newborns allergy (LINA) cohort. Although only trace levels of BBP were detected in e-liquids compared to other phthalates [123], it is of import to note that BBP shares a common metabolite—mono-n-butyl phthalate (MnBP)—with phthalates more abundant in e-liquids, such as diethyl phthalate. Therefore, it is imperative to elucidate whether BBP, MnBP, or other phthalate metabolites induce specific epigenetic modifications. A significant correlation between maternal urinary MnBP levels during pregnancy and asthma symptoms in the child persisting until at least 6 years of age has been reported [126].

Direct e-cigarette vapour exposure leads to impaired innate immune responses in murine lungs [127], whilst murine models of MEV exposure have shown inimical effects of e-cigarette vapour on neonatal lung development [128]. There is a current paucity of studies on the impact of MEV exposure on the foetal epigenome but those that have been published thus far demonstrate that MEV exposure leads to epigenetic aberrations in the offspring. A murine model of MEV exposure with and without nicotine on cognitive function found that exposure to MEV without nicotine significantly increased global DNA methylation in the offspring when compared to ambient air-exposed offspring, whilst MEV with nicotine did not [129]. The study further showed that *DNMT3a* and *DNMT3b* mRNA were ameliorated by MEV without nicotine. Furthermore, mRNA for genes involved in histone modifications *Carm1*, *Atf2*, *Aurka*, *Aurkb* and *Aurkc* were also augmented by MEV without nicotine only. Thereby suggesting that e-cigarette vapour is capable of epigenetic modulation in the offspring independent of nicotine.

An investigation into the impact of MEV exposure on respiratory outcomes found that MEV exposure with and without nicotine induced significant global DNA hypermethylation in offsprings’ lungs compared to air-exposed controls [130]. Interestingly, MEV without nicotine elicited significantly greater DNA hypermethylation compared to those induced by MEV with nicotine with enhanced expression of the pro-inflammatory cytokines IL-5, IL-13, TNF-α mRNA only seen in the lungs of offspring exposed to MEV without nicotine [130]. The analysis of changes in global DNA methylation patterns demonstrates that exposure to MEV is inducing heritable epigenetic changes that manifest in the offspring. Although nicotine-containing e-vapour has been shown to induce less hypermethylation than non-nicotine containing e-vapour, the profile of which genes are being methylated or demethylated is not yet known. Therefore, further investigation is necessary to elucidate where in the genome the modifications are taking place and the roles these genes play in pathophysiology before making a congruent decision on the role of e-vapour with and without nicotine plays in epigenetics and respiratory disease.

Nicotine concentrations in e-liquid in the EU are permitted to be as high as 20 mg/ml; although, some samples exceed that limit [121] leaving users susceptible to higher nicotine exposure than anticipated. Studies on indoor air quality have detected increased levels of nicotine and carcinogenic nitrosamines, such as *N*-nitrosonornicotine (NNN) and nicotine-derived nitrosamine ketone (NNK) in the atmosphere after e-cigarette use [131]. Serum cotinine levels measured in non-smoking and non-vaping individuals exposed to environmental e-vapour found elevated cotinine levels that equated to ETS exposure and persisted at the same rate as ETS [61, 132], suggesting that e-vapour remains in the atmosphere in a similar fashion to ETS. Further, nicotine remaining in the indoor environment can react with oxidant gases in the atmosphere to form added levels of NNN and NNK [133]. Nitrosamines have been shown to methylate DNA and induce methylation DNA damage [134], which is a mechanism believed to be behind their carcinogenicity [135, 136].

Studies have shown that foetal nicotine levels equate to those in the mother [137] with nicotine capable of accumulating in the respiratory tract in the foetus [42]. Animal models of nicotine-only exposure show that offspring exhibit increased smooth muscle and collagen bulk in the airway, and augmented airway hyperreactivity [138–140]. Altered lung development was shown to persist in second-generation offspring not exposed to nicotine [141]. A murine model of nicotine exposure showed that perinatal nicotine exposure altered DNA methylation and histone modification in the lung and gonads of offspring and induced asthma-like changes that persisted into the third generation of offspring [142], thereby demonstrating functional respiratory and epigenetic effects induced by maternal nicotine exposure, together with direct epigenetic changes to the germline. Corroborating with these changes was a decrease in mRNA and protein expression of peroxisome proliferator-activated receptor γ (PPARγ) which plays an essential role in lung development and repair [142–144]. Interestingly, when Rosiglitazone, a known PPARγ agonist, was administered in concert with nicotine to pregnant dams, asthma-like changes and H3 acetylation induced by nicotine exposure was prevented whilst nicotine induced global H4 acetylation and DNA methylation persisted [145], further reinforcing the significance of PPARγ’s role in healthy lung development. These seemingly paradoxical effects of nicotine in e-liquids compared to those described earlier in relation to cigarette smoking may relate to the dose and duration of exposure and to its well-known anti-inflammatory effects [146].

## The future of epigenetic therapeutics

The established role of epigenetics in pathophysiology naturally implores exploring its therapeutic potential. Using 5-azacytidine to inhibit DNMT1 in a murine model of asthma augmented numbers of Treg cells and effectively reduced airway inflammation [147]. The pan-HDAC inhibitor, Trichostatin-A, has similarly shown efficacy in asthma models [148], as has the allosteric activator of SIRT1, SRT1720 [149]. Targeting HDAC classes 1–3 with MS-275 abrogated neutrophil infiltration of the lungs and expression of proinflammatory cytokines KC, IL-6 and IL-1β [150]. An in vitro model of asthma using human airway smooth muscle cells attenuated TGF-β-induced proliferation and pro-inflammatory cytokine production with bromodomain inhibitors JQ1(+) and I-BET762 [151]. Using inhibitors to target proteins and enzymes active in epigenetic modulation are useful tools in demonstrating the effect of certain classes of epigenetic changes. However, due to the nature of their targets, it is difficult to determine the complete extent of which genes are within the purview of the inhibitors.

To overcome this impediment, epigenetic therapeutics may focus on the use of DNA targeting systems capable of binding to genes of interest in a directed manner. The three most well-understood DNA targeting systems are zinc finger proteins (ZFPs), transcriptional-activator-like effectors (TALEs), and clustered regularly interspaced short palindromic repeats (CRISPR) and CRISPR-associated protein 9 (Cas9, 152); the latter of which being the most recent advance in the field and most efficient as it is less cumbersome than ZFPs and TALEs [152]. A study of *SPDEF*—a regulator of mucus production in COPD known to be hypomethylated [153]—in human lung epithelial cells effectively used ZFPs and CRISPR/dCas to attenuate mucus-related gene expression and reduce mucus production by silencing *SPDEF* [154]. Therein demonstrating that targeted silencing of genes using epigenetic editing can reverse disease pathologies in vitro.

## Conclusion

The evidence summarised in this review demonstrates that maternal use of tobacco cigarettes and e-cigarettes and exposure to environmental tobacco smoke induces epigenetic changes in the offspring. These changes have been demonstrated to contribute to disease pathology and be passed down to further generations independent of exposure. The all-encompassing nature of epigenetic modifications implores research to consider using cell types specifically implicated in disease pathologies, as findings across differing cell types may obfuscate pathological epigenetic differences with inherent epigenetic differences dictating cell phenotype. Further, it is imperative to continue exploring intergenerational effects of maternal e-cigarette use and exposure using animal models on DNA methylation at specific genomic regions and specific chromatin modifications to relate the changes being induced to genes implicated in disease pathology, thereby elucidating targets for the use of advanced DNA targeting systems in therapy. Finally, it is recommended that further longitudinal studies on the impacts of e-cigarettes are carried out, thereby allowing us to distinguish between epigenetic modifications that are biomarkers of exposure, such as the aforementioned *CYP1A1* and *AHRR* versus those that are likely to mediate airway disease susceptibility.

## Abbreviations

ASM: Airway smooth muscle
BAL: Bronchoalveolar lavage
BBP: Benzylbutylphthalate
BET: Bromo- and extra-terminal domain
CAS9: CRISPR-associated protein 9
COPD: Chronic Obstructive Pulmonary Disease
CRISPR: Clustered regularly interspaced short palindromic repeats
DNA: Deoxynucleic acid
DNMT: DNA methyltransferase
ETS: Environmental tobacco smoke
EU: European Union
EWAS: Epigenome-wide association study
FEV1: Forced Expiratory Volume in one second
GWAS: Genome-wide association study
HAT: Histone acetyltransferase
HDAC: Histone deacetylase
HDM: House dust mite
IFNγ: Interferon gamma
*Igf1*: Gene for insulin growth factor 1
*Igf1R*: Gene for receptor of insulin growth factor 1
*Igf2*: Gene for insulin growth factor 2
IL: Interleukin
LINA: Lifestyle and environmental factors and their influence on newborns allergy
METS: Maternal exposure to environmental tobacco smoke
MEV: Maternal e-cigarette vapour
miRNA: MicroRNA
MnBP: Mono-n-butyl phthalate
mRNA: Messenger RNA
MTS: Maternal use of tobacco smoke
NNK: Nitrosamine ketone
NNN: *N*-nitrosonornicotine
PAHs: Polycyclic aromatic hydrocarbons
SNP: Single-nucleotide polymorphism
TALEs: Transcriptional-activator-like effector
TGF-β: Transforming growth factor beta
ZFP: Zinc finger protein
